# Protect MSM from HIV and other sexually transmitted diseases by providing mobile health services of partner notification: protocol for a pragmatic stepped wedge cluster randomized controlled trial

**DOI:** 10.1186/s12889-020-09162-x

**Published:** 2020-07-14

**Authors:** Xiangyu Yan, Yongjie Li, Hexuan Su, Yi Xing, Bo Zhang, Zuhong Lu, Zhongwei Jia

**Affiliations:** 1 Department of Epidemiology and Biostatistics, School of Public Health, Peking University, Beijing, China; 2 National Institute on Drug Dependence, Peking University, 38 Xueyuan Rd, Haidian District, Beijing, 100191 China; 3 Medical Informatics Center, Peking University, Beijing, China; 4 School of Public Health, Institute of Child and Adolescent Health, School of Public Health, Peking University, Beijing, China; 5 State Key Laboratory for Bioelectronics, School of Biological Science and Medical Engineering, Southeast University, Nanjing, China; 6 Center for Drug Abuse Control and Prevention, National Institute of Health Data Science, Peking University, Beijing, China; 7 Center for Technology and Policy Research on Infectious Disease Prevention and Control, Global Health Research Institute, Peking University, Beijing, China

**Keywords:** Stepped wedge cluster randomized controlled trial, Partner notification, Men who have sex with men, HIV incidence, Sexually transmitted disease, mHealth application

## Abstract

**Background:**

Recently, more and more men who have sex with men (MSM) look for casual partners through online dating platforms in China. However, most are unable to know their partners’ HIV and other sexually transmitted diseases (STD) statuses, leading to the rapid increase in HIV infection among Chinese MSM. Effective partner notification is urgently needed to increase the risk awareness of MSM and prevent HIV and other STDs transmission. However, the traditional intervention mainly targets to the HIV-positive MSM and the effect is not promising. Our study aims to provide Internet-based partner notification, along with a series of health services for HIV-negative MSM to protect them from HIV and other STDs.

**Methods:**

A pragmatic stepped wedge cluster randomized controlled trial design is used to evaluate the effectiveness of a new intervention paradigm, which aims to reduce HIV and other STDs incidences among MSM in China. Through integrating a mobile health (mHealth) service application (app) to the current HIV and other STDs prevention and control methods, the new paradigm provides partner notification of HIV, syphilis, hepatitis B, and hepatitis C statuses. A total of 6172 MSM in 16 districts of Beijing, China will be recruited and randomized to sequentially receive partner notification intervention through the app at 6-month intervals. The primary outcomes are HIV incidence and the additional cost of the intervention. The secondary outcomes include incidences of syphilis, hepatitis B, and hepatitis C, disease transmission social networks, testing adherence, knowledge of HIV and STDs control, health self-responsibility awareness, changes of high risk behaviors and other related outcomes. The generalized linear mixed models (GLMM) will be used to analyze the differences of outcomes in the control period and in the intervention period.

**Discussion:**

We expect that the HIV incidence will be significantly lower and the secondary outcomes will also be improved with providing health service of partner notification through mhealth intervention. The feasible and affordable public health intervention paradigm will have implications for HIV and STDs prevention and control among MSM and other key populations.

**Trial registration:**

ClinicalTrials.gov, NCT04349748. Registered on 16 April 2020.

## Background

The HIV/AIDS epidemic in China is developing rapidly in recent years, with the number of people living with HIV increasing from 0.44 million in 2011 to 0.95 million in 2019 [1, 2]. Men who have sex with men (MSM) are the major key population of HIV/AIDS. Male homosexual transmission has become the second major HIV transmission route in China only after heterosexual transmission, which accounts for about a quarter of the new HIV/AIDS cases [2, 3]. Having unprotected anal sex is the main risk of MSM to be infected, which is also associated with interrelated risk factors, such as non-persistent sexual partners, alcohol and drug use, and co-infection of other sexually transmitted diseases (STDs) [4–10].

With the development of the Internet and the popularity of mobile devices, the ways and venues of MSM seeking sexual partners had changed dramatically. A change from seeking sexual partners through offline venues, such as gay bars, baths and parks, to online ways through Internet or mobile applications (app) is witnessed in recent years [11–13]. The rise of online dating applications (ie, Blued, Grindr) in China tremendously reduces the cost and inconvenience for MSM to seek casual sexual partners [14–16]. Some MSM who sought sexual partners through the Internet do not know each other before, not to mention each other’s HIV infection status, which brings higher risk of HIV transmissions [17, 18]. In addition, the convenience of transportation increases the risk of cross-regional HIV transmission. All of these lead to greater difficulties in HIV prevention and control among MSM who sought sexual partners through Internet, as it is always difficult to find the sexual partner again afterwards. Recent research in China showed the HIV incidence among MSM online dating apps users was about four times higher than that of nonusers (8.5 per 100 person-years vs 2.0 per 100 person-years) [19]. Therefore, MSM’s behaviors of seeking sexual partners through Internet should be paid more attention. It is important for them to know their sexual partners’ real HIV status, strengthen the awareness of health self-responsibility and give each other the opportunity to make decisions on whether to meet and have sex.

Partner notification is a public health approach to prevent and control HIV infection among HIV high-risk subpopulations, especially MSM. It is recommended by the World Health Organization (WHO) and has been implemented in 67 countries around the world, including China [20]. Partner notification denotes that the sexual partners should take the initiative to tell each other their HIV infection statuses, especially those living with HIV/AIDS; it also encourages sexual partners to receive HIV testing [20]. Most existing methods of partner notification target to newly HIV-infected individuals’ sexual partners, for example, health workers may provide assisted HIV partner notification services on the condition that the newly HIV-infected individuals agree to, through personal telephone or address contact, e-postcards, and some website-based methods [21–23]. However, these methods aimed to offer HIV-infected individuals an opportunity to tell their sexual partners about their HIV statuses anonymously after sexual behaviors, which is a afterward remedy. Partners of the HIV-infected individuals are still at high risks. Meanwhile, some issues on the feasibility and effectiveness of these existing partner notification methods exist, including difficulties in daily implementation, low response rate, and high cost in time, human resources etc. [24, 25].

Seeing these problems, we developed a mobile health (mHealth) app for partner notification to encourage interactive queries among MSM before having sex, even before meeting. It targets not only to HIV-infected MSM, but HIV negative MSM as well. Through integrating the mHealth app to the current HIV and other STDs prevention and control methods, we set up a new and feasible public health intervention paradigm, to realize the goal of moving forward the intervention and reduce the HIV incidence among MSM.

This study has several objectives. First, we aim to evaluate the effect of the additional partner notification intervention in reducing HIV incidence among MSM. Second, the incremental cost of the added interventions based on existing HIV prevention measures is analyze, to evaluate whether it is cost-effective. Our third objective is to evaluate the effect of the additional partner notification intervention in some secondary outcomes, including syphilis and other sexually transmitted diseases incidence, HIV and related diseases transmission among social networks, HIV testing adherence, knowledge and health self-responsibility awareness, changes of sexual behaviors, etc. We hypothesize that the intervention services are effective and cost-efficient.

## Methods/design

### Study design and timeline

**Fig. 1:**
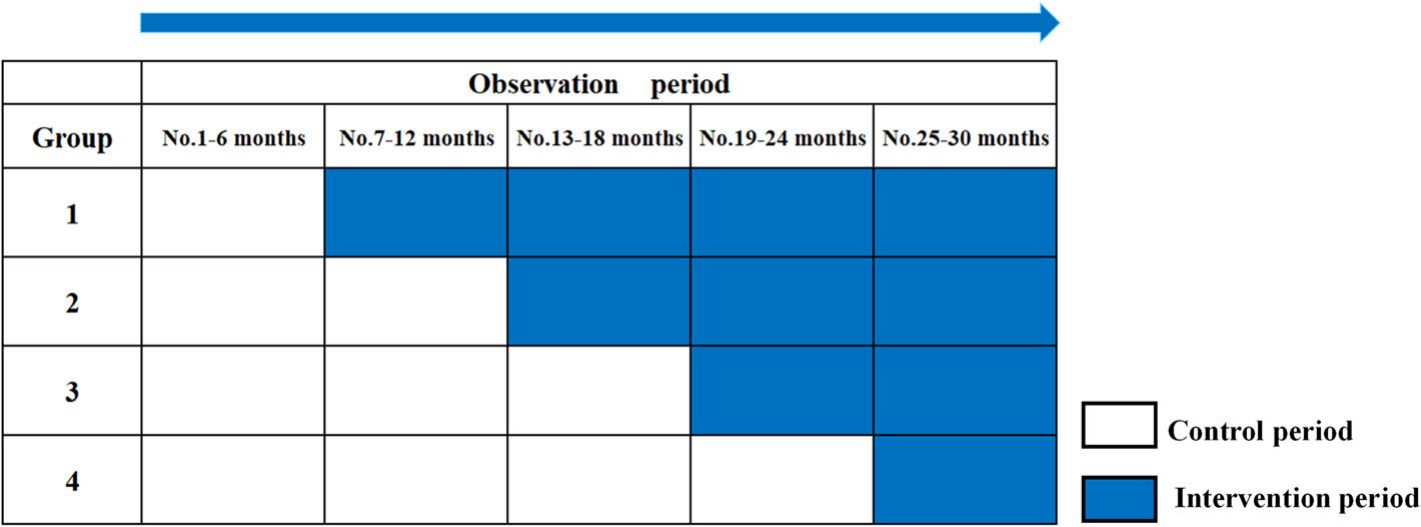
Stepped wedge design of the intervention implementation

This research will use a stepped wedge cluster randomized controlled trial (SW-CRCT) design. It is commonly used to evaluate service delivery or policy interventions of clusters [26]. This research will be implemented in Beijing, whose sixteen districts are defined as the clusters. We assign the sixteen districts into four research groups using stratified randomization, with four districts in one group. The open cohort SW-CRCT will last 2.5 years, which is anticipated to be conducted from July, 2020 to December, 2022. Five continuous observational periods at a 6-month interval are divided. According to the standard design of SW-CRCT, in the first 6-month “the initial period”, no group is exposed to the additional partner notification intervention. After that, the four groups begin to cross from the control condition to receive the partner notification intervention sequentially at 6-month intervals. Once one group receives the partner notification intervention, the intervention will continue until the end of the research. And at the last 6-month period, all of the four groups will receive the additional partner notification intervention. (Fig. 1).

There are several reasons for such a SW-CRCT design. Firstly, the implementation of partner notification needs communication and interaction among MSM, which can only be achieved through the accumulation of a larger number of participants at the cluster (district) level after the recruitment period. Therefore, the intervention needs to be applied to cluster-level instead of individual-level, and cluster randomization is used instead of individual randomization. Secondly, SW-CRCT design is a pragmatic design that is increasingly used worldwide in recent years. This design allows to evaluate the effect of intervention in a real-world condition considering the social environment and logistical issues, which aligns with our aims [26, 27]. Thirdly, the SW-CRCT design ensures that all clusters and participants receive the partner notification intervention, which is more ethical considering the partner notification and other interventions we implement may not bring obvious harm to our participants [26, 28].

### Study setting and recruitment

We will conduct our research in Beijing, the capital city of China with high population mobility and demographic heterogeneity and high burden of HIV and related STDs among MSM. We will cooperate with a local community-based organization (CBO) named *Man Wellness Center* to recruit participants and provide HIV and other STDs (syphilis, hepatitis B, and hepatitis C) testing services. *Man Wellness Center* is one of the largest and most popular CBO for MSM to give them HIV and STDs prevention and medical services.

Participants will be recruited in two ways: offline and online. The offline recruitment will be implemented in offline venues, such as the clinic sites of the CBO for routine work, and the gay bars, bathrooms, parks for outreach services. The online recruitment will be implemented through online platforms, such as WeChat, QQ, Weibo and Blued. The process of recruitment includes four steps: (1) Determine whether the participant recruited meets the eligibility criteria. (2) Introduce the research and tools for interventions (the app and the WeChat official account) if eligible. (3) Help the participant sign the electronic informed consent through the app and then show him the functions of the app and WeChat official account. The content of the electronic informed consent contains all necessary information as traditional paper version of informed consent, and the signed electronic informed consent will be stored in the database. Participants under the age of 18 are asked to register the app system and sign the consent accompanied by a guardian, and written consent is also obtained from the guardian. (4) Invite the participant to fill in the baseline questionnaire through the app. (5) Provide HIV and other STDs testing services and upload the test results through the backend database. The recruitment will start from the beginning of the research, and we will stop recruitment in the last 6 months of the research. If the participant has HIV and other STDs testing during the follow-up period, he will be invited to finish a follow-up questionnaire.

If the participant is recruited offline, to protect the participants’ privacy and confidentiality, the process of the recruitment will be implemented one-to-one by the CBO’s trained staff in a private environment. If the participant is recruited online, the process will also be implemented one-to-one through online platforms. After finishing the baseline questionnaire, the participant will be suggested to use the HIV and STDs self-testing services or go to the CBO clinics to receive facility-based testing. If the online recruited participant does not conduct the testing 1 month after registering, he will receive testing reminding information through the app and mobile phone short message.

Ethical Approval for this study has been obtained from the Peking University Institutional Review Board (IRB00001052–16016).

### Eligibility and exclusion criteria for participants

Eligibility criteria of participants in this research include: (1) biologically male, (2) have oral or anal sex with men at least once during their lifetime, (3) aged 15 years or older, (4) have no difficulty using a mobile phone, (5) willing to provide their mobile phone numbers to serve as the unique identification numbers of the app’s self-query and partner notification functions, (6) willing to use the app’s function modules, (7) willing to complete the questionnaire for the research, (8) willing to continuously receive HIV and STDs testing and consulting services provided by the CBO our research setting at, and (9) willing to complete the informed consent document. MSM who had serious physical disabilities or mental diseases will be excluded.

MSM who meet all the eligibility criteria, but whose first HIV test results are positive, will also be invited to register as app users and complete the questionnaire. However, they will not be included in the statistical analysis of HIV incidence and other outcomes except for social network related outcomes.

### Interventions

#### Tools of interventions

The tools of interventions contain a WeChat official account and an app with four functional modules centering on partner notification.

The WeChat official account is developed with three functions. First, it can be used as a service platform to provide the online “Mailing rapid test reagent kit” self-testing service. Participants can make an appointment of HIV and STDs rapid test reagent kit through it. Second, health education materials can be sent to participants through the WeChat notification messages weekly. Third, it can be a medium for participants logging in the app. We set a link to the app at the WeChat official account, so participants can easily and quickly to use the functional modules of the app. (Additional Fig. 1).

The app we develop for this research named *Golden ark*, which contains four functional modules, including partner notification module, test result self-query module, prompt and warning module, and health education module. The functions of these modules are as follows [29].

### Partner notification module

**Fig. 2:**
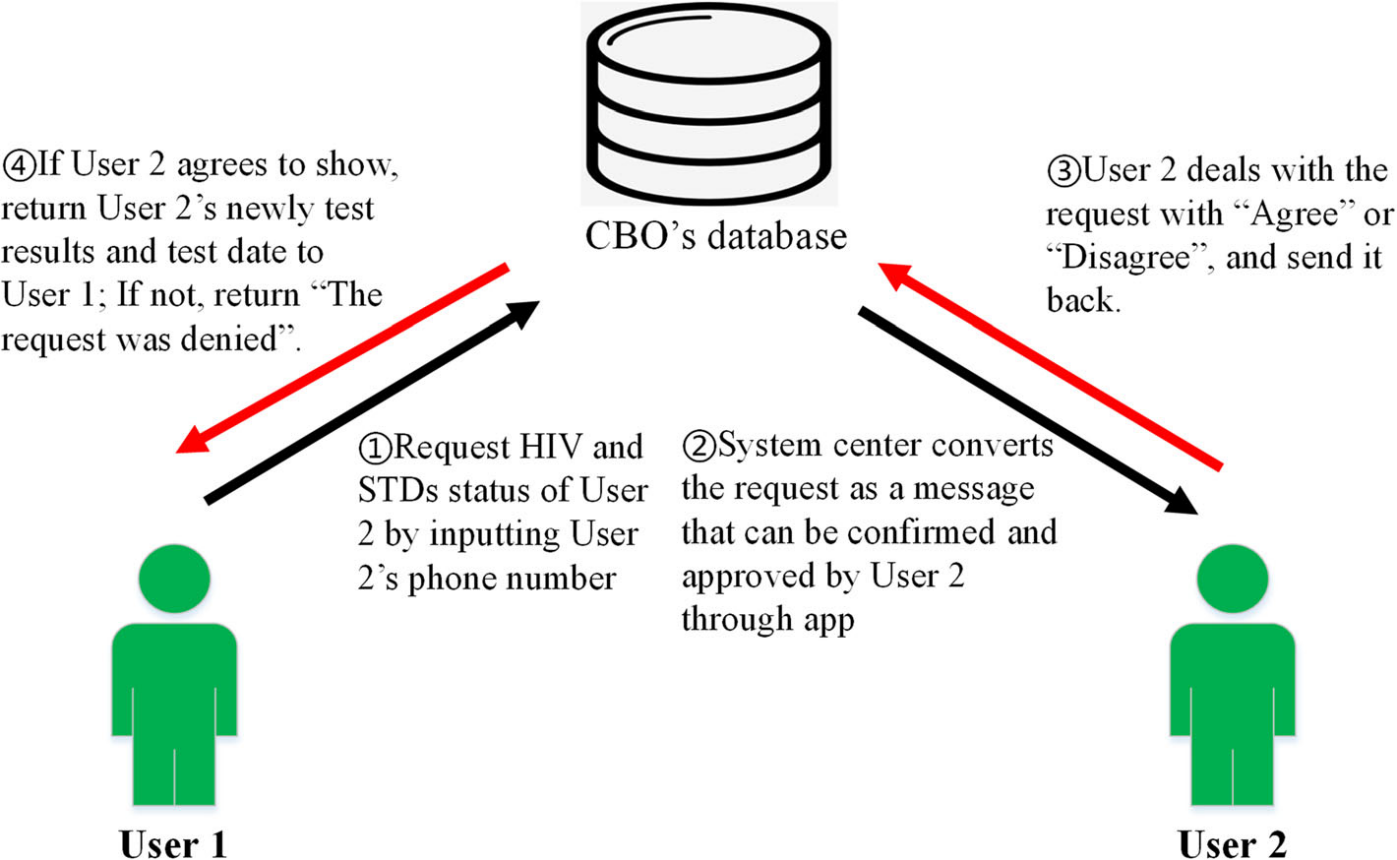
The framework of the partner notification

This module allows users to request each other’s recent HIV and other STDs status and the test date from the CBO’s database. To be ethical and privacy-protecting, the applicant should be permitted by those the applicant requests for. The framework of the partner notification (User 1 requests the status of User 2) contains four steps: (1) User 1 requests User 2’s HIV and other STD’s status by using User 2’s phone number through the app. (2) System center (CBO’s database) will convert the request as a message that can be confirmed and approved by User 2 through App. (3) User 2 deals with the request with “Agree” or “Disagree”, and send it back to the system center. (4) If User 2 agrees to inform User 1, system center will return User 2’s recent HIV and other STD’s test results and the date of the testing to User 1. If not, return “The request was denied”. (Fig. 2, Additional Fig. 2).

### Test result self-query module

After completing HIV and STD testing, CBO’s staff will upload the test results and dates into the database, so users can query their own test results and dates through the app’s test result self-query module. (Additional Fig. 3).

### Prompt and warning module

**Fig. 3:**
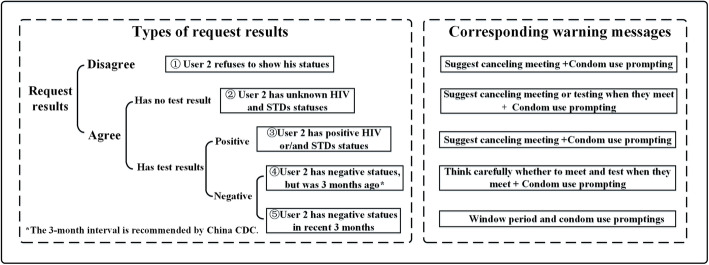
Types of request results and corresponding warning messages

In addition to some fixed prompts and warnings for information security concern, this module also provides some dynamic prompts and warnings based on their own test records and the responses of partner notification they received. For their own test records, the users will receive prompts to encourage them to have HIV and STDs testing monthly through app as well as mobile phone short messages according to the dates of their last test records. For partner notification, based on the User 2’s response and infection status shown as follows, the app will send different prompts and warnings to protect User 1 from the potential risks. (Fig. 3, Additional Figs. 2, 3).

### Health education module

In this module, health education materials will be sent to participants weekly through the WeChat official account. An additional function of this module is that the history records of health education materials can be classified and saved in the module, so users can review the materials when they need any time. (Additional Fig. 4).

#### Intervention implementation

Based on the CBO’s existing HIV and STD prevention methods, such as HIV and STDs testing services, we construct a new integrated intervention model for HIV and STDs control among MSM by adding two parts of interventions.

The first part is to strength MSM’s safety awareness of seeking sexual partners and sense of health self-responsibility. Two measures will be implemented as follows: (1) Provide health education materials weekly to participants through the WeChat official account and app’s health education module. These materials contain the harmfulness of HIV/AIDS and STDs, symptoms and signs of HIV/AIDS and STDs, awareness of receiving HIV and STDs testing on their own initiative, awareness of partner notification, HIV/AIDS and STDs prevention skills, related laws, related drug use instruction, and the harmfulness of drug abuse and its prevention. The content of health education will repeat every 6 months in order to ensure all participants receive the whole education content. (2) Provide HIV and STDs testing prompting service to HIV negative participants monthly according their test records individually through app’s prompt and warning module and mobile phone short messages.

The second part is to promote and encourage partner notification through the app. We expect if two MSM are dating through some online dating platforms, the partner notification and related warnings provided via the app will in a timely manner help them know each other’s HIV and STDs status before they are going to meet and have sex.

This research is designed as a pragmatic SW-CRCT study. The interventions participants received in the control period and the intervention period are different. In control period, they will receive health education through the WeChat official account and app, as well as HIV and STDs testing prompting service. The app’s test result self-query module is also opened to them to promote the use of the app and avoid the loss to follow-up. But the partner notification is not available. In intervention period, all interventions in the control period will continue, and the partner notification functions will be opened. (Fig. 1).

### Randomization and allocation

The randomization is conducted in cluster (district) level, instead of individual level. The previous records of the CBO show that most MSM receiving HIV testing services are from the following four districts: Chaoyang, Shunyi, Fengtai, and Haidian. Considering the number balance of participants in each groups, stratified randomization is used. The process of randomization is conducted by a researcher using SAS software: (1) Assign each district a number, A1 to A4 for the four districts that the research may recruit more potential participants (Chaoyang, Shunyi, Fengtai and Haidian), B1 to B12 for other twelve districts. (2) Extract without putting it back one number in districts A and three numbers in districts B to be one sampling group. Finally, the sixteen districts will be randomized into four intervention groups. (3) Randomize the order of four intervention groups to be Group 1 to 4, and the additional partner notification intervention using app will be implemented to these groups in sequence every 6-month intervals. The participants will be assigned to different groups according to the district where they live recorded in the baseline questionnaire.

As the partner notification is a kind of behavioral intervention, it is impossible that participants do not know what intervention they receive. So, the research is only single-blind to outcome assessors. However, we take efforts to reduce the information bias caused by the non-blind design of other stakeholders. At the beginning of the research, participants, staff of the CBO, and the researchers who participate in management of participants will not know the randomized scheme of the research. The additional partner notification will be opened to participants sequentially by the predetermined computer program, which does not need CBO staffs and researchers to implement the intervention manually. The CBO staffs and researchers do not know the implementation of partner notification unless the participants tell them whether they can use the partner notification function of the app on their own initiative. Even the CBO staff knows that, the operation and results of the HIV and STDs testing will not be affected. Also, the outcomes assessors do not know the randomized scheme in the process of data analysis.

### HIV and STDs testing

HIV and STDs testing will be conducted at baseline investigation during participants recruitment, and follow-up investigations by CBO staffs. There are two alternative ways that participants can receive testing services, including the “Mailing rapid test reagent kit” self-testing service and the facility-based testing service.

**Fig. 4:**
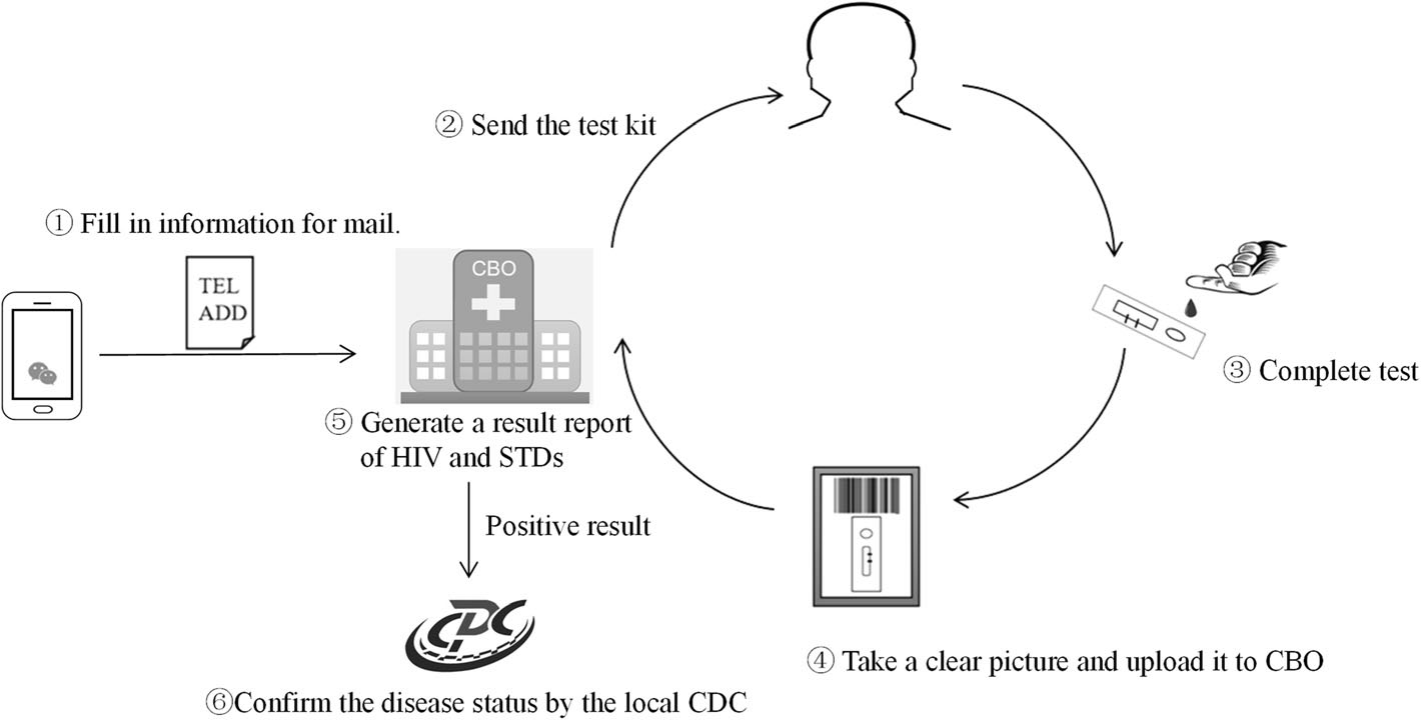
Flow chart of the “Mailing rapid test reagent kit” testing service. (The images were drawn by the author team of this study)

The “Mailing rapid test reagent kit” self-testing service bases on participants’ applications on the WeChat official account. The procedures for one participant applying for HIV and STD self-testing are as followed: (1) Fill in phone number and address information used to mail the test kit through the WeChat official account; (2) The CBO will send a package of rapid test reagent kit to the user (the kit is free of charge, but the express fee charged by express company is required); (3) Complete the self-testing using the test kit by himself. If he does not know how to use, he can see a teaching video provided on the WeChat official account; (4) Take a clear picture of the test reagent kit and upload it to CBO through the system; (5) CBO’s trained staff will check the results and generate a testing report for the user showing the result “Negative” or “Positive” of HIV and STDs and the date of this testing service. (Fig. 4).

For the facility-based testing service, when one participant comes to the clinic sites of the CBO for testing, the testing service will be implemented one-to-one by the CBO’s trained staff. After testing, the CBO’s staff will input the test results and date into the backend database, and the participant can get an electronic test result report.

In addition, if a participant’s HIV or other STDs’ test result is positive, the CBO staffs will contact him, help him confirm the disease status by referring him to the local Centers for Disease Control and Prevention (CDC) and provide him additional health care. The test reagent kit used in both “Mailing rapid test reagent kit” self-testing service and the facility-based testing service will be the HIV, HCV, TP, HBsAg quaternary Colloidal Gold reagent (Wondfo^@^).

### Sample size

We used the *steppedwedge* procedure in Stata software set for SW-CRCT design to calculate the sample size. We assumed the HIV incidence was 7% in the control status, and could be reduced to 5% after partner notification intervention. The parameters set for sample size calculation include: (1) The number of clusters was 16; (2) The number of steps was 4, with 4 clusters in one step; (3) α was set as 0.05; (4) Power was set as 0.90; (5) ICC (ρ) was set as 0.01. The total sample was 4320 (270 for each clusters). Considering most studies of MSM showed a high proportion of loss to follow up, we set a 30% loss to follow up in this research. The total sample size was 6172.

### Quality control

Half a month before the implementation of the research, researchers of Peking University will conduct training to CBO staffs in following aspects: (1) the usage of the “Golden ark” app’s function modules, (2) participants recruitment process, (3) HIV and STDs testing methods, (4) answers of frequently asked questions, (5) work quality requirements. After completing the training and passing the test, staff of CBO will be given a staff IDs and conduct the participants recruitment work.

During the implementation of the research, quality control will include three parts: quality control conducted by CBO, quality control conducted by researchers, and quality control conducted by expert committee.

Quality control conducted by CBO includes: (1) The leader of the CBO should check whether the participants’ information records are complete and correct every 2 weeks. The focus is on whether the recruited participants meet the inclusion criteria and have completed the informed consent and the baseline questionnaire. (2) The leader of the CBO should check at least 10 cases of HIV and STDs testing services every month, to see whether the testing operation and input of test results are correct. The above quality control implementation should be recorded and submitted to researcher of Peking University for inspection. (3) Record any harms reported by participants, including psychological and emotional problems and any privacy information disclosure risk, and then report these harms to the research team timely.

For quality control conducted by researchers, it includes the following three aspects: (1) Perform regular backups of data in the database, including HIV and STDs testing records, questionnaire records and queries records. (2) Provide weekly feedbacks to CBO on the list of participants with incomplete information through information system, and respond to the reported harms. (3) Go to the study sites of CBO every 3 months, and conduct spot checks on at least 5% of the staff’s operation, including participants recruitment, HIV and STDs testing services. Interviews with three to five participants will be conducted each time to know more about the issues in the research process.

The expert committee will conduct meetings every 6 months, who will review the annual data analysis reports, the interim analysis reports and reports of potential harms to identify the problems in the research and propose corrective actions. If the effectiveness of the intervention is already significant before the endpoint of this 2.5-years trial, or unexpected harms have been caused to participants, the committee will discuss and make the decision of whether to terminate the trial.

### Outcomes

The primary outcomes of this research are the HIV positive seroconversion rate (HIV incidence) and the additional cost of the intervention. The HIV positive seroconversion rate is calculated as the number of HIV positive seroconversions divided by the total number of person-years. The additional cost of the intervention is calculated as the total cost and average cost for per participant of the integrated intervention model and each additional intervention/service based on the existing HIV prevention measures provided as before.

The second outcomes of this research include: (1) Positive seroconversion rate (incidence) of syphilis, hepatitis B, and hepatitis C. These indicators are calculated separately as the number of syphilis/hepatitis B/hepatitis C seroconversions divided by the total number of person-years. (2) HIV and related STDs transmission among social networks. Based on the partner notification information, we will construct the transmission networks of HIV and other STDs (syphilis, hepatitis B, and hepatitis C) among participants, and analyze the characteristics of these networks and factors associated with transmission of these diseases. (3) Testing adherence. This indicator is calculated as the proportion of participants whose every adjacent testing interval is no more than 3 months as recommended by China CDC. (4) Additional cost for finding per seroconversion. This indicator is calculated through the total additional cost of the integrated intervention model based on the existing HIV prevention measures provided as before divided by the total number of the HIV and STDs seroconversions. (5) Frequency of utilization for each intervention/service. This indicator is calculated through the total and average frequency of utilization for each intervention/service, such as health education, partner notification, HIV and STDs testing and so on. (6) Satisfaction of the interventions and services, measured through the follow-up questionnaires by the degree of satisfaction for participants of HIV and STDs testing service, health education, partner notification and other app’s functions. (7) Knowledge of HIV and related STDs. This indicator is measured by the baseline and follow-up questionnaires. The score of knowledge questions in the questionnaires will be used to represent participants’ level of knowledge. (8) Health self-responsibility awareness, including attitudes of HIV and STDs testing, safe sexual behaviors and partner notification. The attitudes are collected by questions in the baseline and follow-up questionnaires. (9) Sexual behaviors. They are measured through questionnaires, including questions about the number of different kinds of sexual partners, frequency of anal sex, condom use, and partner notification implementation. (10) Substance use behaviors. This indicator is measured by questionnaires about participants’ usage of methamphetamine, heroin, ecstasy, and some other sex-promoting drugs. (11) Psychological status. Self-rating anxiety scale (SAS) and Self-rating depression scale (SDS) are used to measure participants’ psychological status through questionnaires. (12) Referral rate of HIV and related STDs. This indicator is calculated as the proportion of new HIV and STDs infections who have received treatment. (Additional file 2).

### Data management

We developed a data-management platform integrated with the WeChat official account and the app for partner notification. Data are from three sources: (1) HIV and STDs statuses and dates of tests approved and submitted to the management system by the CBO’s staff; (2) baseline and follow-up questionnaires; (3) self-query behaviors (self-query records) and the interactive behaviors (partner notification records) of app users. Participants will be linked through the three date sets by a unique ID, which will be a 16-bit string made up of random letters and numbers transformed from their registered phone number to ensure participants’ privacy.

To protect data security, the data are stored in an encrypted database by a stand-alone server in case of information leakage and illegal use. Hierarchical permissions for CBO’s staff and researchers are set to restrict the viewing and exporting the data. When exporting and analyzing data, all the data will be anonymized. We also set up an independent data management advisory committee to review and evaluate the process of data collection, database management, data analysis, data sharing, and study results.

### Analysis

Descriptive analysis will be used to summarize the socio-demographic characteristics, HIV and STDs incidences, as well as knowledge, attitudes and related behaviors of total participants and different subgroups. To investigate the effects of the additional partner notification intervention, we will compare the differences of these primary and secondary outcomes in the control period and in the intervention period. As the HIV and STDs infection and most of the secondary outcomes are categorical variables, the generalized linear mixed models (GLMM) will be used for analysis. The model will include intervention status (in intervention period or not) and time as fixed effects and clusters (the districts) and individuals as random effects. The estimated effects of partner notification will be reported as risk ratio (RR) for binary outcomes and mean difference for continuous outcomes. 95% confidential intervals (CI) and *p* values will also be reported. The total and average additional costs of the intervention will be calculated to reflect the cost-effectiveness of the additional interventions, and the differences of these indicators in the control period and intervention period will be compared. Based on the connections recorded in the partner notification records of the database, social network visualization will be conducted to reflect the real connections of the participants. Social network analysis, such as calculation of degree, density, clique, degree centrality, closeness centrality, betweenness centrality, clustering coefficient, and so on, will be used to describe the characteristics of the social networks. Several transmission models of HIV and STDs will be formed to reflect the diseases transmission among MSM.

Sub-analysis will include: comparison of the effects in different age groups, different sex roles (insertive only, receptive only, both), different usage of HIV and STDs testing services (“Mailing rapid test reagent kit” only, the facility-based only, both). A sensitivity analysis will be conducted to evaluate the cluster (the district)-level effect. We will use the outcomes of each district across the follow-ups as the dependent variable, and fit a linear mixed model with the random effect of clusters (the districts).

If one variable is missing for < 10% of participants, we will use a complete-case approach. If one variable is missing for 10 ~ 20% of participants, multiple imputation will be used if suitable; if one variable is missing for > 20% of participants, we will investigate the mechanism of missing data, and consider to set the missing data as a category.

A two-sided *p* value of 0.05 or less was regarded as significant. Statistical analyses will be done with SPSS version 21.0, Stata version 24.0, R version 3.5.1. Social network visualization will be done with Cytoscape version 3.5.1.

The results of this research will be reported to local and national stakeholders in the form of oral presentation on professional conferences and publications on journals. We will not use professional writers in writing related articles and reports. The decisions of authorship will follow conventions of the International Committee of Medical Journal Editors. We will share our study protocol, statistical analysis plan, informed consent form, study report and analytic code after completing the analysis of the whole study. If other researchers want to apply for the above information and data of this research, they should contact principal investigator. Personal information (name, unit, title, etc.), detailed description of required data (required variables, etc.), and research proposal are needed for further discussion and decision.

## Discussion

One important reason that HIV keeps increasing among MSM in China is that most MSM seeking causal sexual partners through online dating platforms (such as Blued and Zank), as it results in much more risk sexual behaviors for HIV and other STDs transmission [30, 31]. Partner notification of HIV and STDs infection statuses before they decide to meet and have sex can be an important and feasible method to reduce the HIV and STDs transmission among sexually active MSM. China’s Internet penetration rate has reached 61.2% by June 2019, and 99.1% of Internet users use mobile phones to surf the Internet [32]. The intervention tools based on the WeChat-the most popular mobile phone social software [33], and a website-based app enable the method more acceptable, convenient and intimate for MSM. It may lead to better effects compared with the existing partner notification methods provided by health workers individually or through email cards. An observational study using the previous version of this app has shown a potential positive effect of the app-based partner notification intervention in reducing HIV infection among MSM [29]. An encouraging finding was that the longer the app was used, the lower the HIV incidence (> 5 months vs ≤ 5 months: 2.22 per 100 person-years vs 6.99 per 100 person-years; risk ratio 0.32, 95% CI 0.12–0.87), and the app has also been proved to be user-friendly and could be accepted and widely used by MSM [29]. However, the blank control without receiving the partner notification intervention was lacking in the observational study. Meanwhile, the observation period was only 1 year and the sample size was small, so the effectiveness of the intervention needs to be further evaluated by this trial.

In this research, we create a feasible and affordable public health intervention paradigm, based on the existing HIV and STDs control methods. We integrate health education to strengthen their safety awareness and health self-responsibility, HIV and STDs testing prompting service and partner notification through app platform. To promote the convenience and privacy of HIV and STDs testing, we innovatively adopt multiple forms of testing service including the newly online HIV self-testing method “Mailing rapid test reagent kit” and the facility-based service. It can give participants more choices and may improve the adherence of the research. In this research, the SW-CRCT design is used for ethical and logistical concerns, which can minimize bias and make sure all participants receive the partner notification intervention. We expect our partner notification intervention can reduce the incidence of HIV and other related STDs, and it is cost-effective to be widely popularized in HIV and STDs control activities. It may also improve MSM’s knowledge, health self-responsibility awareness of HIV and STDs prevention, and testing adherence, as well as to reduce high risk behaviors. Based on the partner notification records we collect through the app, an innovative social network analysis will be conducted. The networks formed by the real partner notification connections are more objective and comprehensive than the connections found by traditional observational or cohort studies. The characteristics of the social networks can provide more guidance for HIV and STDs control among MSM.

One limitation of this research is that even though the interventions based on Internet and mobile phone app can be easily accepted by youth participants, some elderly people might feel it difficult to use. But it is worth noting that recent researches have showed that the age of recruited MSM is mainly 25 to 40 years old.

Our research is the first study to conduct Internet- and app-based integrated partner notification intervention in a large simple size, and will generate important research and policy implications for HIV and STDs control among MSM.

### Trial status

At the time of writing this protocol article, participants recruitment and data analysis of this research has not begun. The research is registered in the ClinicalTrials.gov database (NCT 04349748). The database will also be used for documenting protocol modifications. The trial protocol conforms to the Standard Protocol Items: Recommendation for Interventional Trials (SPIRIT) 2013 statement.

## Supplementary information

**Additional file 1: Figure S1.** Interfaces of the WeChat official account’s home page and HIV self-testing service. **Figure S2.** Interface of *Partner notification* module and the warnings in this module. **Figure S3.** Interface of *Test result self-query* module and the prompt in this module. **Figure S4.** Interface of *Health education module*

**Additional file 2.** Questionnaire (English version)

## Data Availability

No applicable.

## Abbreviations

MSM: Men who have sex with men
mHealth: Mobile health
app: Application
STD: Sexually transmitted diseases
GLMM: Generalized linear mixed models
WHO: World Health Organization
SW-CRCT: Stepped wedge cluster randomized controlled trial
CBO: Community-based organization
CDC: Center for Disease Control and Prevention
SAS: Self-rating anxiety scale
SDS: Self-rating depression scale
