# Supplementary material for: Protect MSM from HIV and other sexually transmitted diseases by providing mobile health services of partner notification: protocol for a pragmatic stepped wedge cluster randomized controlled trial
Source: BMC Public Health. 2020 Jul 14;20:1107. doi: 10.1186/s12889-020-09162-x (PMC7362655; doi:10.1186/s12889-020-09162-x)
Supplement: Supplementary file 1 — Additional file 1: Figure S1. Interfaces of the WeChat official account’s home page and HIV self-testing service. Figure S2. Interface of Partner notification module and the warnings in this module. Figure S3. Interface of Test result self-query module and the prompt in this module. Figure S4. Interface of Health education module [file 12889_2020_9162_MOESM1_ESM.docx]

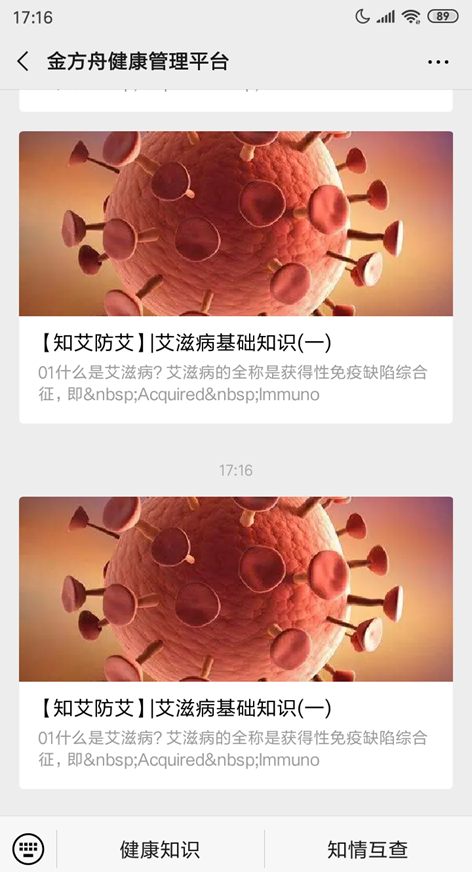

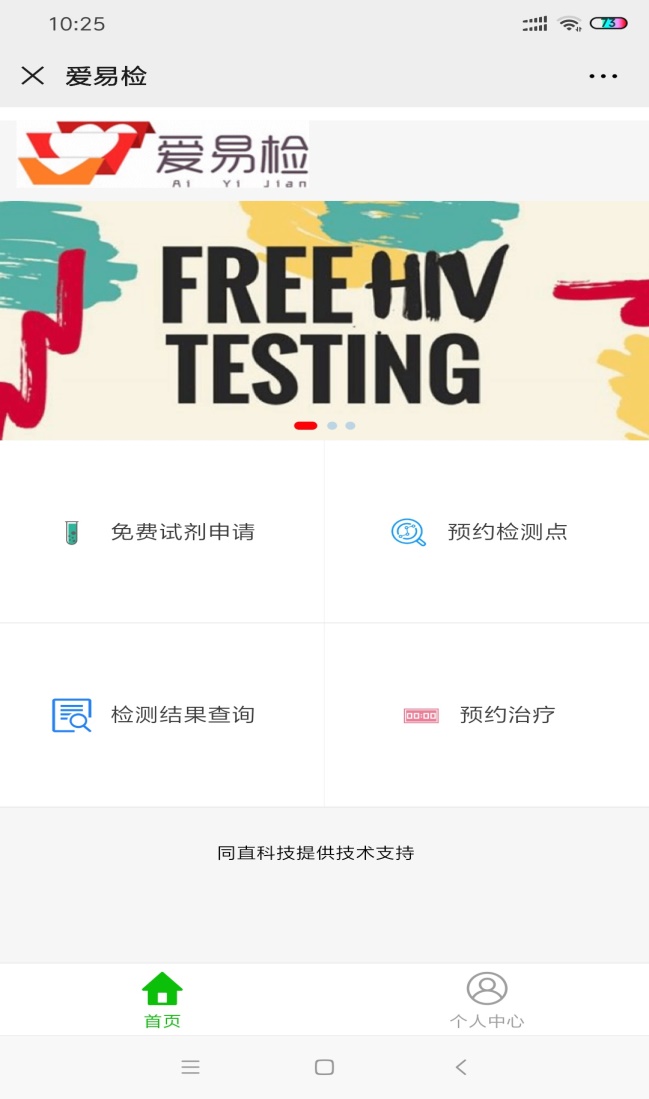


(a) Home page of the WeChat official account (b) HIV testing service

**Additional Fig. S1 Interfaces of the WeChat official account’s home page and HIV self-testing service**


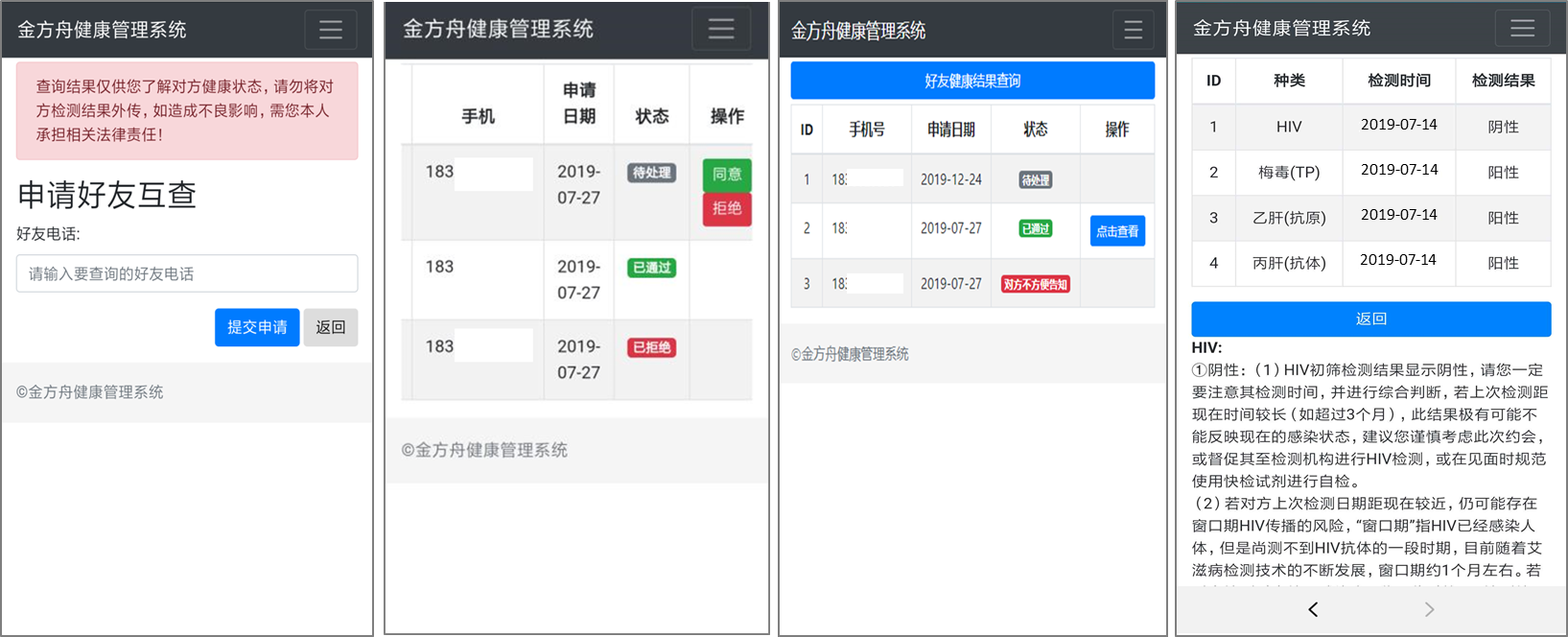


(a) Send request (User 1) (b) Deal with the request (User 2) (c) Receive processing results (User 1) (d) See User 2’s testing results and

warnings sent by the system

**Additional Fig. S2 Interface of *Partner notification module* and the warnings in this module.**


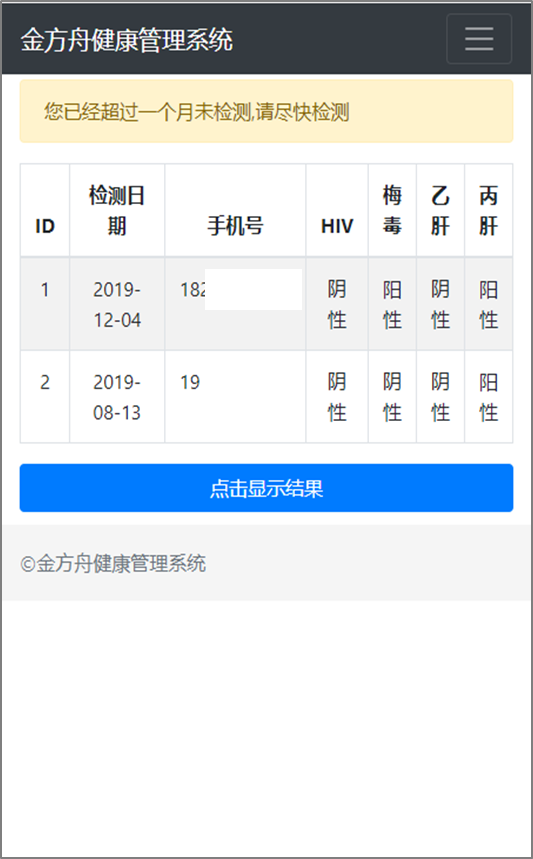


**Additional Fig. S3 Interface of *Test result self-query* *module* and the prompt in this module.**


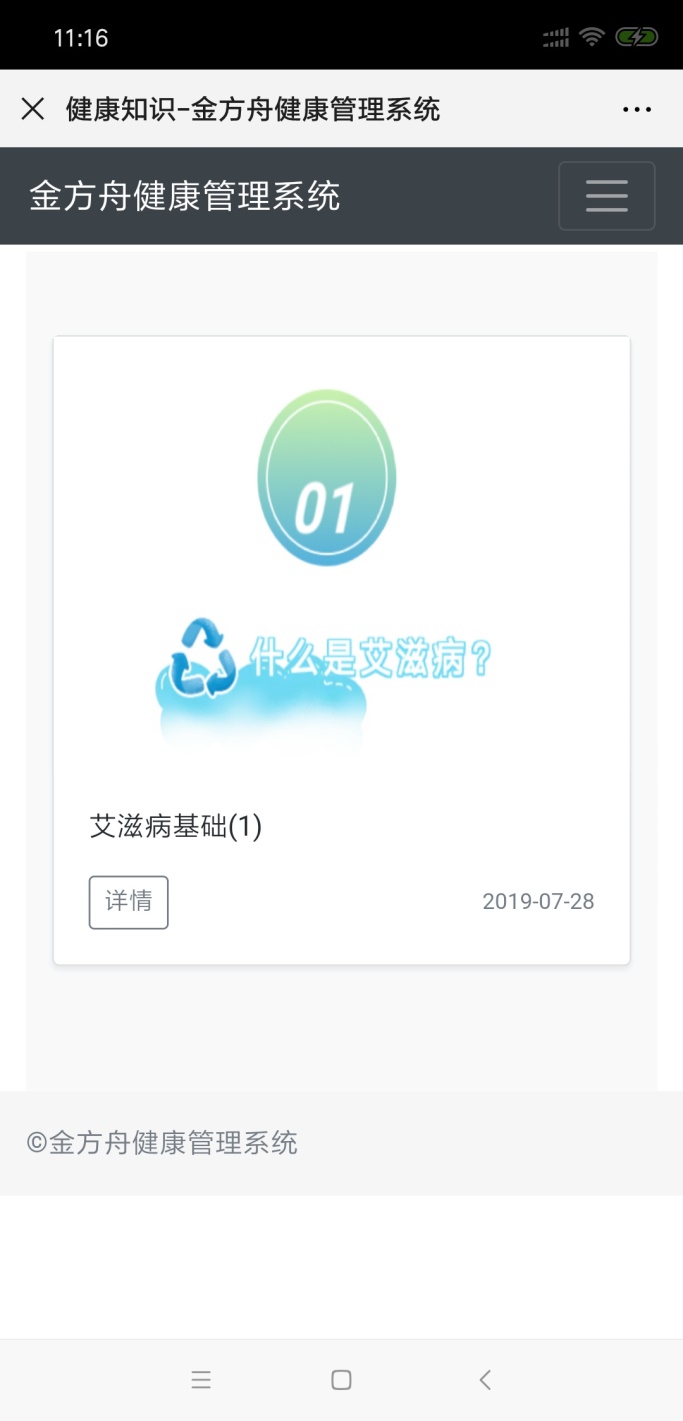


**Additional Fig. S4 Interface of *Health education module***
