# Supplementary material for: Protect MSM from HIV and other sexually transmitted diseases by providing mobile health services of partner notification: protocol for a pragmatic stepped wedge cluster randomized controlled trial
Source: BMC Public Health. 2020 Jul 14;20:1107. doi: 10.1186/s12889-020-09162-x (PMC7362655; doi:10.1186/s12889-020-09162-x)
Supplement: Supplementary file 2 — Additional file 2. Questionnaire (English version) [file 12889_2020_9162_MOESM2_ESM.docx]

**Survey Questionnaire (English version)**

**A. Sociodemographics**

**A01. Birthday：** (Month/year) (For example, 11/1995)

**A02. Gender identity:** ① Male ② Female

**A03. What is your sexual orientation?**

①Homosexual ②Heterosexual ③Bisexual ④Unsure/Other

**A04. Nationality:** (For example, Han)

**A05. Current marital status:**

①Unmarried and live alone

②Unmarried but live with boy/girl friend

③Married

④Separated or divorced or widowed

**A06. Domicile place:**

①Beijing

②Other provinces, China [which province: ]

③Other countries [which country: ]

**A07. Residence time in Beijing:**

①< 3 months ②3~6 months ③7~12 months ④1 ~2 years ⑤> 2 years

**A08. Which district of Beijing is your present address in?**

①Dong cheng ②Xi cheng ③Chao yang ④Feng tai ⑤Shi jing shan

⑥Hai dian ⑦Men tou gou ⑧Fang shan ⑨Tong zhou ⑩Shun yi

⑪Chang ping ⑫Da xing ⑬Huai rou ⑭Ping gu ⑮Mi yun ⑯Yan qing

**A09. The highest level of education:**

①High school or below

②Junior high school

③High school or vocational school

④Bachelor or associate degree

⑤Above bachelor’s degree

**A10. What is your occupation?**

①Student ②Labor worker ③Farmer/herdsman/fisherman ④Cadre/ civil servant

⑤Teachers, or technicians of research institutions ⑥Medical worker ⑦Soldier

⑧Business/service worker ⑨Unemployed ⑩Other [which occupation______]

**A11. What is your total individual monthly income from all sources? [For students, fill in the amount of money received each month]:_______**RMB

**A12. Your height is:** ________cm

**A13. Your weight is:** ________kg

**B. Knowledge**

**B01. Where do you usually search for or obtain HIV/AIDS related knowledge? [select all that apply]**

①Publicity of medical institutions ②School education ③Family education

④Publicity of community

⑤Education of public security bureau or compulsory detoxification

⑥Publicity by other organizations, such as LGBT NGOs

⑦Through the Internet, such as websites, apps ⑧Newspapers or books

⑨Other: ⑩Never search for or obtain HIV/AIDS related knowledge

**B02. Do you think HIV/AIDS is a serious and incurable infectious disease?**

①Yes ②No ③I do not know

**B03. Do you think men who have sex with men are the most affected by HIV/AIDS in China?**

①Yes ②No ③I do not know

**B04. Do you think you can tell if a person is infected with HIV/AIDS by their appearance？**

①Yes ②No ③I do not know

**B05. Do you think contracting other STDs increases the risk of HIV/AIDS infection?**

①Yes ②No ③I do not know

**B06. Do you think that consistent and correct use of condoms reduces the risk of contracting and spreading HIV?**

①Yes ②No ③I do not know

**B07. Do you think the use of drugs (methamphetamine, ecstasy, ketamine, etc.) increases the risk of HIV infection?**

①Yes ②No ③I do not know

**B08. Do you think HIV testing and counseling should be actively sought after high-risk behaviors (needle sharing, unsafe sex, etc.)?**

①Yes ②No ③I do not know

**B09. Do you think the intentional spread of HIV should bear legal responsibility?**

①Yes ②No ③I do not know

**C. Attitudes related to sexual health**

**Do you agree with the following behaviors or opinions?**

**[Choose only ONE attitude for each question]**

**C01. Always use condoms when having anal sex.**

①Strongly agree ②Agree ③No specific view ④Disagree ⑤Strongly Disagree

**C02. Taking the initiative to test for HIV regularly and other STDs using self-test reagents or going to the appropriate institution is a sign of responsibility.**

①Strongly agree ②Agree ③No specific view ④Disagree ⑤Strongly Disagree

**C03. Before having sex, one should proactively inform his sexual partner if he is infected with HIV or other STDs.**

①Strongly agree ②Agree ③No specific view ④Disagree ⑤Strongly Disagree

**C04. Before having sex, one should ask his sexual partners if they are infected with HIV or other STDs.**

①Strongly agree ②Agree ③No specific view ④Disagree ⑤Strongly Disagree

**Do you think the infection of HIV/AIDS will bring certain harm to the following aspects of the infected person?**

| **Harm** | **①No** | **②Mild** | **③Moderate** | **④Severe** |
| --- | --- | --- | --- | --- |
| **C05. Physical impairment** |  |  |  |  |
| **C06. Mental health impairment** |  |  |  |  |
| **C07. Harm of Family relationships** |  |  |  |  |
| **C08.Harm of careers** |  |  |  |  |
| **C09.Harm of reputation** |  |  |  |  |

**C10.** **Would you like to go to a testing facility such as an LGBT NGO for an on-site HIV and STD test?**

①Strongly willing to ②Willing to ③No specific view

④unwilling to ⑤Strongly unwilling to

**C11.** **Would you like to accept the “Mailing rapid test reagent kit”** **self-testing service provided by the LGBT NGO? (You need to apply for rapid test reagent online, and the reagents will be delivered to you soon. After you conduct self-testing, you need to take a photo of the test reagent kit and upload it through the system, and the staff of the institution will review the results and provide you with an electronic version of the result report)**

①Strongly willing to ②Willing to ③No specific view

④unwilling to ⑤Strongly unwilling to

**C12. Do you think that if a person receives the “Mailing rapid test reagent kit” service and the results are positive, he is willing to take a photo and upload it back?**

①Strongly willing to ②Willing to ③No specific view

④unwilling to ⑤Strongly unwilling to

**C13.** **How much do you think you are at risk of being infected with HIV?**

①No ②Low ③General ④Relatively high ⑤Very high

**D. Sexual behaviors**

**D01. Have you ever had sex with a person?**

①Yes ②No (Skip to D28)

**D02. The biological sex of the sexual partners with whom you have had sex:**

①Male only ②Female only (Skip to D24) ③Both male and female

**D03. How old were you when you first had sex with a male?** __________years

**D04. Which of the following forms of sexual activity have you first had with a male? [select all that apply]**

①Oral sex ②Anal sex ③Masturbate for each other ④Other:___________

**D05. The reason you first had sex with a male: [select all that apply]**

①Love him and willing to have sex with him ②Because of curiosity

③Be forced to ④Because of the money paid after sex

⑤Seduced by other ways other than money

⑥The pursuit of pleasure and excitement ⑦Other：_______________

**D06. Your relationship with your first male sexual partner:**

①Close friend ②Ordinary classmate or colleague ③Boyfriend

④Casual sexual partner with money transaction

⑤Casual sexual partner without money transaction (“one-night stand”)

⑥Other situation：___________

**D07. Where do you usually seek male sexual partners？**

①Bar/dance hall ②Teahouse/clubhouse ③Public bathroom

④Park/public toilet/lawn ⑤Internet or software app ⑥Other venue:_______

**D08. Which of the following types of sexual partners have you had homosexual anal sex with so far? [select all that apply]**

①Sexual partner with money transaction

②Regular sexual partners without money transaction

③Casual sexual partners without money transaction (“one-night stand”)

④Never have homosexual anal sex (Skip to D24)

**D09. What is your sex role when you have homosexual anal sex?**

①Receptive only(0) ②Insertive only(1) ③Both(0.5)

**D10. Have you ever had condomless sex with HIV infected male (including condom loss during sex)?**

①Yes ②No ③I do not know

**D11. In the past 3 months, which of the following types of sexual partners have you had homosexual anal sex with? [select all that apply]**

①Sexual partner with money transaction (If choose this, answer questions D12-D15)

②Regular sexual partners without money transaction (If choose this, answer questions D16-D19)

③Casual sexual partners without money transaction (“one-night stand”) (If choose this, answer questions D20-D23)

④Never have homosexual anal sex (Skip to D24)

| **D12. In the past 3 months, how many sexual partners with money transaction have you had homosexual anal sex with?**  The number is:___________  **D13. In the past 3 months, how often do you use condoms when you have homosexual anal sex with** **sexual partners with money transaction?**  ①Every time ②Sometimes ③Never  **D14. In the past 3 months, have you taken the initiative to inform sexual partners your true HIV and STDs statuses when you have homosexual anal sex with sexual partners with money transaction?**  ①Inform all of the sexual partners ②Inform some of the sexual partners  ③Do not inform to any sexual partner  **D15. In the past 3 months, do you know your sexual partners’** **HIV and STDs statuses when you have homosexual anal sex with sexual partners with money transaction?**  ①All sexual partners are known ②Some of sexual partners are known  ③Do not know disease statuses of any sexual partner |
| --- |
| **D16. In the past 3 months, how many regular sexual partners without money transaction have you had homosexual anal sex with?**  The number is:___________  **D17. In the past 3 months, how often do you use condoms when you have homosexual anal sex with** **regular sexual partners without money transaction?**  ①Every time ②Sometimes ③Never  **D18. In the past 3 months, have you taken the initiative to inform sexual partners your true HIV and STDs statuses when you have homosexual anal sex with regular sexual partners without money transaction?**  ①Inform all of the sexual partners ②Inform some of the sexual partners  ③Do not inform to any sexual partner  **D19. In the past 3 months, do you know your sexual partners’** **HIV and STDs statuses when you have homosexual anal sex with regular sexual partners without money transaction?**  ①All sexual partners are known ②Some of sexual partners are known  ③Do not know disease statuses of any sexual partner |
| **D20. In the past 3 months, how many casual sexual partners without money transaction have you had homosexual anal sex with?**  The number is:___________  **D21. In the past 3 months, how often do you use condoms when you have homosexual anal sex with** **casual sexual partners without money transaction?**  ①Every time ②Sometimes ③Never  **D22. In the past 3 months, have you taken the initiative to inform sexual partners your true HIV and STDs statuses when you have homosexual anal sex with casual sexual partners without money transaction?**  ①Inform all of the sexual partners ②Inform some of the sexual partners  ③Do not inform to any sexual partner  **D23. In the past 3 months, do you know your sexual partners’** **HIV and STDs statuses when you have homosexual anal sex with casual sexual partners without money transaction?**  ①All sexual partners are known ②Some of sexual partners are known  ③Do not know disease statuses of any sexual partner |

**D24. In the past 3 months, which of the following drugs have you used ? [select all that apply]**

①Methamphetamine ②Heroin ③Ketamine ④Ecstasy ⑤RUSH

⑥ “Ling hao” capsule ⑦The G-spot liquid ⑧LSD ⑨Marijuana

⑩opium ⑪Cocaine ⑫Sleeping pills (used without a doctor's prescription)

⑬Magu ⑭Other：____________ ⑮Never used (Skip to D26)

**D25. In the past 3 months, have you used drugs before or during sex to enhance your sexual experience?**

①Often used ②Occasionally used ③Never used

**D26. In the past 3 months, have you had group sex?**

①Yes ②No

**D27. In the past 3 months, have you had sex with female?**

①Yes ②No

**D28. Have you been tested for HIV before?**

①Yes, [frequency:________times/year] ②No(Skip to D30)

**D29. Where have you been tested for HIV before? [select all that apply]**

①Hospital ②VCT clinic of CDC ③LGBT NGO’s clinic

④In parks, bars and other recreational places to accept on-site testing services

⑤Buy rapid test reagent for self-testing in drugstores and other places

⑥Apply to LGBT NGO for“Mailing rapid test reagent kit” self-testing service through WeChat or other apps

⑦Other：___________

**D30. Have you been tested for syphilis before?**

①Yes, [frequency:________times/year] ②No(Skip to D32)

**D31. Where have you been tested for syphilis before? [select all that apply]**

①Hospital ②VCT clinic of CDC ③LGBT NGO’s clinic

④In parks, bars and other recreational places to accept on-site testing services

⑤Buy rapid test reagent for self-testing in drugstores and other places

⑥Apply to LGBT NGO for“Mailing rapid test reagent kit” self-testing service through WeChat or other apps

⑦Other：___________

**D32. Have you been tested for HBV before?**

①Yes, [frequency:________times/year] ②No(Skip to D34)

**D33. Where have you been tested for HBV before? [select all that apply]**

①Hospital ②VCT clinic of CDC ③LGBT NGO’s clinic

④In parks, bars and other recreational places to accept on-site testing services

⑤Buy rapid test reagent for self-testing in drugstores and other places

⑥Apply to LGBT NGO for“Mailing rapid test reagent kit” self-testing service through WeChat or other apps

⑦Other：___________

**D34. Have you been tested for HCV before?**

①Yes, [frequency:________times/year] ②No(Skip to D36)

**D35. Where have you been tested for HCV before? [select all that apply]**

①Hospital ②VCT clinic of CDC ③LGBT NGO’s clinic

④In parks, bars and other recreational places to accept on-site testing services

⑤Buy rapid test reagent for self-testing in drugstores and other places

⑥Apply to LGBT NGO for“Mailing rapid test reagent kit” self-testing service through WeChat or other apps

⑦Other：___________

**D36. In the past 3 months, have you taken PrEP drugs?**

①Yes ②No(Skip to D38)

**D37. In the past three months, do you adhere to take PrEP drugs as prescribed by doctor?**

①Yes ②No

**D38. In the past three months, have you taken PEP drugs?**

①Yes ②No

**D39. Which of the following services have you used to communicate with your sexual** partner about HIV and STDs status?

①You two go to the testing institutions for testing, and exchange testing results

②Inform your sexual partners anonymously by health works in the testing institutions

③Other services or software:__________

④Never used any service

**D40. Have you ever used gay dating apps such as Blued or Zank, or read posts or websites that discuss gay topics?**

①Yes ②No(Skip to D42)

**D41. What are your reasons for using the gay dating apps, forums or websites? [select all that apply]**

①Just out of curiosity, to get to know about gays

②Learn HIV/AIDS prevention and other related knowledge

③Make friends, find gym mates, etc.

④Seek sexual partners

⑤Drug trafficking (methamphetamine, etc.)

⑥Other:______________________

**D42. Have you ever engaged in any of the following behaviors? [select all that apply]**

①Property related adverse events such as theft and fraud

②Affray, picking quarrels and provoking troubles

③Cause damage to the natural environment (illegal logging, illegal mining, damage to environmental resources protection, etc.)

④Obstruction of official duties (obstruction of the execution of official duties, destruction of flammable and explosive equipment, etc.)

⑤Traffic accidents (traffic accidents, damage to traffic facilities, etc.)

⑥Crimes related to sex (rape, obscenity, whoring, etc.)

⑦Other:___________

⑧No these behaviors

**E. Health conditions**

**Have you ever suffered from or received medical treatment for the following diseases?**

**E01. Genital inflammation**

①Never have an examination for the disease

②After examination, I am free from the disease

③After examination, I suffered from the disease and received medical treatment

④After examination, I suffered from the disease, but did not receive medical treatment

**E02. Genital/perianal/oral herpes**

①Never have an examination for the disease

②After examination, I am free from the disease

③After examination, I suffered from the disease and received medical treatment

④After examination, I suffered from the disease, but did not receive medical treatment

**E03. Hepatitis B**

①Never have an examination for the disease

②After examination, I am free from the disease

③After examination, I suffered from the disease and received medical treatment

④After examination, I suffered from the disease, but did not receive medical treatment

**E04. Hepatitis C**

①Never have an examination for the disease

②After examination, I am free from the disease

③After examination, I suffered from the disease and received medical treatment

④After examination, I suffered from the disease, but did not receive medical treatment

**E05. Genital/perianal/oral condyloma**

①Never have an examination for the disease

②After examination, I am free from the disease

③After examination, I suffered from the disease and received medical treatment

④After examination, I suffered from the disease, but did not receive medical treatment

**E06. Genital chlamydia trachomatis infection**

①Never have an examination for the disease

②After examination, I am free from the disease

③After examination, I suffered from the disease and received medical treatment

④After examination, I suffered from the disease, but did not receive medical treatment

**E07. Gonorrhea**

①Never have an examination for the disease

②After examination, I am free from the disease

③After examination, I suffered from the disease and received medical treatment

④After examination, I suffered from the disease, but did not receive medical treatment

**E08. Syphilis**

①Never have an examination for the disease

②After examination, I am free from the disease

③After examination, I suffered from the disease and received medical treatment

④After examination, I suffered from the disease, but did not receive medical treatment

**E09. Tuberculosis**

①Never have an examination for the disease

②After examination, I am free from the disease

③After examination, I suffered from the disease and received medical treatment

④After examination, I suffered from the disease, but did not receive medical treatment

**For each item below, please check the column which best describes how often you felt or behaved this way during the past several days.**

|  | 1. A Little Of The Time | 2. Some Of The Time | 3. Good Part Of The Time | 4. Most Of The Time |
| --- | --- | --- | --- | --- |
| E10. I feel more nervous and anxious than usual. |  |  |  |  |
| E11. I feel afraid for no reason at all. |  |  |  |  |
| E12. I get upset easily or feel panicky. |  |  |  |  |
| E13. I feel like I’m falling apart and going to pieces. |  |  |  |  |
| E14. I feel that everything is all right and nothing bad will happen. |  |  |  |  |
| E15. My arms and legs shake and tremble. |  |  |  |  |
| E16. I am bothered by headaches neck and back pain. |  |  |  |  |
| E17. I feel weak and get tired easily. |  |  |  |  |
| E18. I feel calm and can sit still easily. |  |  |  |  |
| E19. I can feel my heart beating fast. |  |  |  |  |
| E20. I am bothered by dizzy spells. |  |  |  |  |
| E21. I have fainting spells or feel like it. |  |  |  |  |
| E22. I can breathe in and out easily. |  |  |  |  |
| E23. I get numbness and tingling in my fingers and toes. |  |  |  |  |
| E24. I am bothered by stomach aches or indigestion. |  |  |  |  |
| E25. I have to empty my bladder often. |  |  |  |  |
| E26. My hands are usually dry and warm. |  |  |  |  |
| E27. My face gets hot and blushes. |  |  |  |  |
| E28. I fall asleep easily and get a good night’s rest. |  |  |  |  |
| E29. I have nightmares. |  |  |  |  |

**For each item below, please check the column which best describes how often you felt or behaved this way during the past several days.**

|  | 1. A Little Of The Time | 2. Some Of The Time | 3. Good Part Of The Time | 4. Most Of The Time |
| --- | --- | --- | --- | --- |
| E30. I feel down hearted and blue. |  |  |  |  |
| E31. Morning is when I feel the best. |  |  |  |  |
| E32. I have crying spells or feel like it. |  |  |  |  |
| E33. I have trouble sleeping at night. |  |  |  |  |
| E34. I eat as much as I used to. |  |  |  |  |
| E35. I still enjoy sex. |  |  |  |  |
| E36. I notice that I am losing weight. |  |  |  |  |
| E37. I have trouble with constipation. |  |  |  |  |
| E38. My heart beats faster than usual. |  |  |  |  |
| E39. I get tired for no reason. |  |  |  |  |
| E40. My mind is as clear as it used to be. |  |  |  |  |
| E41. I find it easy to do the things I used to. |  |  |  |  |
| E42. I am restless and can’t keep still. |  |  |  |  |
| E43. I feel hopeful about the future. |  |  |  |  |
| E44. I am more irritable than usual. |  |  |  |  |
| E45. I find it easy to make decisions. |  |  |  |  |
| E46. I feel that I am useful and needed. |  |  |  |  |
| E47. My life is pretty full. |  |  |  |  |
| E48. I feel that others would be better off if I were dead. |  |  |  |  |
| E49. I still enjoy the things I used to do. |  |  |  |  |

**F. Satisfaction survey [This part is only used in follow-ups]**

**F01. In the past three months, which of the following services and the app system functions have you acquired or used?**

①The facility-based testing and consulting service (If choose this, answer questions F02-F04)

②“Mailing rapid test reagent kit” self-testing service (If choose this, answer questions F05-F07)

③App’s “Partner notification” module (If choose this, answer questions F08-F10)

④App’s “Test result self-query” module (If choose this, answer questions F11-F13)

⑤App’s “Prompt and warning” module (If choose this, answer questions F14-F16)

⑥WeChat official account/App’s “Health education” module (If choose this, answer questions F17-F19)

⑦None of the above services and functions are used (Skip to F20)

| **F02. What do you think of the facility-based testing and consulting service? [Choose only ONE attitude]**  ①Very satisfied ②Satisfied ③No specific view ④Dissatisfied ⑤Very Dissatisfied  **F03. Are you willing to continue acquiring the facility-based testing and consulting service in the future?**  ①Yes ②No  **F04.Any questions and suggestions for the facility-based testing and consulting service:**____________________________________ |
| --- |
| **F05. What do you think of “Mailing rapid test reagent kit” self-testing service? [Choose only ONE attitude]**  ①Very satisfied ②Satisfied ③No specific view ④Dissatisfied ⑤Very Dissatisfied  **F06. Are you willing to continue acquiring “Mailing rapid test reagent kit” self-testing service in the future?**  ①Yes ②No  **F07.Any questions and suggestions for “Mailing rapid test reagent kit” self-testing service:**____________________________________ |
| **F08. What do you think of app’s “Partner notification” module? [Choose only ONE attitude]**  ①Very satisfied ②Satisfied ③No specific view ④Dissatisfied ⑤Very Dissatisfied  **F09. Are you willing to continue using app’s “Partner notification” module in the future?**  ①Yes ②No  **F10.Any questions and suggestions for app’s “Partner notification” module:**____________________________________ |
| **F11. What do you think of app’s “Test result self-query” module? [Choose only ONE attitude]**  ①Very satisfied ②Satisfied ③No specific view ④Dissatisfied ⑤Very Dissatisfied  **F12. Are you willing to continue using app’s “Test result self-query” module in the future?**  ①Yes ②No  **F13.Any questions and suggestions for app’s “Test result self-query” module:**____________________________________ |
| **F14. What do you think of app’s “Prompt and warning” module? [Choose only ONE attitude]**  ①Very satisfied ②Satisfied ③No specific view ④Dissatisfied ⑤Very Dissatisfied  **F15. Are you willing to continue using app’s “Prompt and warning” module in the future?**  ①Yes ②No  **F16.Any questions and suggestions for app’s “Prompt and warning” module:**____________________________________ |
| **F17. What do you think of WeChat official account/App’s “Health education” module? [Choose only ONE attitude]**  ①Very satisfied ②Satisfied ③No specific view ④Dissatisfied ⑤Very Dissatisfied  **F18. Are you willing to continue using WeChat official account/App’s “Health education” module in the future?**  ①Yes ②No  **F19.Any questions and suggestions for WeChat official account/App’s “Health education” module:**____________________________________ |

**F20. What is your overall view of the services provided by this program? [Choose only ONE attitude]**

①Very satisfied ②Satisfied ③No specific view ④Dissatisfied ⑤Very Dissatisfied

**F21.Any additional questions and suggestions:** _____________________________
